# Fatty acid-binding protein 4 circulating levels in non-segmental vitiligo^☆^

**DOI:** 10.1016/j.abd.2021.04.014

**Published:** 2021-11-25

**Authors:** Azza Gaber Antar Farag, Eman A.E. Badr, Asmaa El-Shafey Soliman El-Shafey, Mustafa Elsayed Elshaib

**Affiliations:** a Andrology and S.T.Ds, Faculty of Medicine, Menoufia University, Shebin AlKom, Egypt; b Medical Biochemistry and Molecular Biology Department, Faculty of Medicine, Menoufia University, Shebin AlKom, Egypt; c Dermatology, Ministry of Health, General Hospital, Shebin AlKom, Egypt; d Faculty of Medicine, Menoufia University, Shebin AlKom, Egypt

**Keywords:** Fatty acid-binding proteins, Metabolic syndrome, Vitiligo

## Abstract

**Background:**

Vitiligo is an acquired and progressive mucocutaneous disease resulting from the loss of active epidermal melanocytes. Metabolic syndrome (MetS) affects about 25% of the world’s population and is linked to inflammatory skin diseases including vitiligo. Fatty Acid-Binding Protein 4 (FABP4) is an intracellular lipid chaperone. FABP4 is closely associated with MetS.

**Objectives:**

To evaluate the serum level of FABP4 in vitiligo patients and its relation to MetS in the investigated cases.

**Methods:**

This case control study was conducted on 45 patients having non segmental vitiligo and 45 matched controls. Their lipid profile, blood glucose and serum FABP4 levels were measured.

**Results:**

There were significant elevations in FABP4 (p < 0.001), cholesterol (p < 0.001), triglycerides (p = 0.005), and glucose (fasting [p = 0.001] and 2 hours post prandial [p < 0.001]) levels in patients in comparison with controls. MetS was significantly more prevalent among vitiligo patients (p < 0.001) and associated with high FABP4 serum levels (p = 0.037). In vitiligo patients, there were significant positive correlations between FABP4 serum levels and triglycerides (p = 0.047), cholesterol (p = 0.001) and LDL (p = 0.001) levels and negative correlation regarding HDL level (p = 0.009). FABP4 level was a significantly good diagnostic test for early detection of vitiligo (p < 0.001).

**Study limitations:**

The small number of studied subjects.

**Conclusions:**

FABP4 may play an active role in the disease process of vitiligo that could be mediated through associated dyslipidemia and hyperglycemia. FABP4 may be a marker of vitiligo helping in its early diagnosis, but it does not appear to be useful for determining vitiligo severity, activity or associated MetS.

## Introduction

Vitiligo is an acquired and idiopathic progressive mucocutaneous disease characterized by damage of working epidermal melanocytes. In nearly half of the patients, vitiligo develops before the age of 20 years however it can be also seen at any age with a non-significant sex difference.^1^

Vitiligo was categorized as Segmental Vitiligo (SV) and Non-Segmental Vitiligo (NSV). In NSV genetic factors of increased risk to autoimmunity were discovered by genome-wide study.^2^ Regulation of innate immune response plus B cell differentiation as well as its activation was demonstrated in NSV,^3^ and were reported to be more prominent in NSV than SV.^4^

Fatty Acid-Binding Protein 4 (FABP 4) is a member of a family of 14–15 kDa proteins, known as intracellular lipid chaperones. They regulate lipid trafficking in cells.^5^ The FABP4, also termed adipocyte protein 2, is formed of 132 amino acids. It represents around 1% of all soluble proteins in adipose tissue.^6^

FABP4 has the ability to bind reversibly to hydrophobic ligands, such as unsaturated and saturated long-chain fatty acids as well as eicosanoids and other lipids protecting organisms against their harmful accumulation.^7^ FABP4 is secreted from adipocytes and macrophages. It is closely associated with obesity and MetS.^8^

MetS is a condition in which insulin resistance is developed and eventually leads to cardiovascular problems.^9^ Around 25% of the population all over the world is affected by MetS, with a substantial subpopulation linked to many inflammatory skin diseases^10^ including vitiligo.^11^

Zhang et al.^12^ found that there were high levels of systemic and local FABP4 in osteoarthritic patients. In the field of dermatology, Baran et al.^13^ reported that serum FABP4 levels were significantly increased in patients with psoriasis. As vitiligo is considered one of the inflammatory diseases, the authors proposed that, in vitiligo, FABP4 may act at the interface of inflammatory and metabolic pathways. Therefore, the aim of this study was to evaluate the serum level of FABP4 in vitiligo patients and its relation to MetS.

## Patients and methods

This is a case-control study that was conducted on 90 subjects. They were 45 patients having different clinical types of NSV and 45 age and gender-matched healthy subjects (control group). They were selected from Dermatology Outpatient Clinic. The diagnosis of vitiligo was made on the basis of the patient’s history and the typical clinical features (discrete, well-circumscribed, de-pigmented macules and patches).

The study was approved by the Committee of Human Rights of Research in the present study’s University. An informed written consent form was obtained from every participant or his and/or her parent (if <18 years) prior to initiation of this study.

The present study included NSV cases from both sexes. Any included patient should stop any systemic (6 weeks) or topical (2 weeks) treatment of vitiligo.

Subjects having immune-inflammatory cutaneous (e.g., psoriasis, atopic dermatitis) and/or systemic (e.g., thyroid, and connective tissue) diseases, and those receiving systemic corticosteroids and/or other immune suppressants within the last one month were excluded from this study.

The studied patients were subjected to history. The Body Mass Index (BMI) was calculated by dividing the body mass (kilograms) by the square of the body height (meters).^14^ Measurement of arterial blood pressure (ABP) was done using a standard mercury sphygmomanometer after the subjects had rested at least for 10 minutes with the arm at heart level.

The dermatological examination was done to ensure the diagnosis of NS vitiligo, determine its site and identify its type (acrofacial, mucosal, generalized, universal, and mixed). Disease severity and activity were evaluated using Vitiligo Area Scoring Index (VASI) and Vitiligo Disease Activity (VIDA) scoring.

To calculate the VASI score, the body was divided into 5 regions: the hands, upper extremities (excluding hands), trunk, lower extremities (excluding the feet), and feet. The axillae were included with the upper, while the buttocks and inguinal regions were included with the lower extremities. One hand unit (the palm plus the volar surface of all digits) was used as a guide to estimate the percentage of vitiligo involvement (1%) of each body region. Depigmentation within each area was estimated regarding the following percentages: 0 (normal pigmented skin), 10% (specks of depigmentation), 25% (pigmented area exceeds the depigmented area), 50% (depigmented and pigmented areas are equal), 75% (depigmented area exceeds the pigmented area), 90% (specks of pigment) and 100% (no pigment). VASI was calculated using this formula: VASI = Σ (all body sites) (hand units) × (depigmentation).^15^

VIDA score was based on the patient's own opinion of his/her disease activity over time. Active vitiligo includes either extension of existing skin lesions or appearance of new ones. The score was graded from +4 (active in the past 6 weeks), +3 (active in the past 3 months), +2 (active in the past 6 months), +1 (active in the past 1 year), 0 (stable for at least 1 year) to −1 (stable for at least 1 year and spontaneous repigmentation).^16^

After overnight fasting, the skin over the vein was sterilized with 70% alcohol, and then 5 mL of venous blood was withdrawn from every subject. Three mL of venous blood was transferred into the plain tube, left to stand for a half-hour, and then centrifuged for 10 min at 4000 R.P.M. The serum was obtained for the determination of lipid profile and FABP4. While 2 mL of blood were transferred into sodium fluoride-containing tubes, centrifuged for 10 min at 4000 R.P.M. The plasma was obtained for the determination of fasting glucose. Another blood sample was obtained for determination of 2 h postprandial glucose. The samples were kept frozen at −20 °C till analysis.

Lipid profile including Triglycerides (TG), cholesterol level, and lipoproteins as High-Density Lipoproteins (HDL) and Low-Density Lipoproteins (LDL), in addition to fasting and 2 hours postprandial blood sugars were measured by an automatic chemistry analyzer (AU480 system from Beckman Coulter, USA).

Diagnosis of MetS was done according to evaluated TG, LDL, FBS level, and blood pressure as follows; TG ≥ 150 mg/dL, HDL-C level <40 mg/dL in men, or <50 mg/dL in women, blood pressure ≥130/85 mm. Hg and fasting hyperglycemia (glucose level >100 mg/dL.^17^

Serum FABP4 was analyzed using Enzyme-Linked Immunosorbent Assay (ELISA) (kit, Quantikine® ELISA, R&D Systems, Inc., USA & Canada).

## Results

### Personal and clinical data of the studied subjects

The included 45 patients were 20 (44.4%) females and 25 (55.6%) male. Their age ranged from 11 to 65 years. They had BMI ranged from 19 to 37 kg/m^2^. Their systolic and diastolic blood pressure ranged from 100 to 150 and from 60 to 90 mmHg, respectively. Patients and the control group were matched as regards their ages, sex, systolic and diastolic blood pressure (p > 0.05 for all). However, BMI was significantly higher in patients than controls (p < 0.001) (Table 1).

**Table 1:** Personal and clinical data of the studied subjects.

Demographic data	Patients (n = 45)	Controls (n = 45)	Test of significance	p-value
Sex				
Male	25 (55.6)	27 (60.0)	χ^2^ = 0.18	0.670
Female	20 (44.4)	18 (40.0)		
Age (years)				
Mean ± SD	35.51 ± 15.98	32.89 ± 13.56	*U* = 0.84	0.404
Median	34	30		
Range	11–65	14–65		
BMI (kg/m^2^)				
Mean ± SD	29.53 ± 5.02	23.89 ± 2.40	*t* = 6.80	<0.001^a^
Median	29	24		
Range	19–37	20–28		
SBP (mmHg)				
Mean ± SD	119.78 ± 15.15	117.56 ± 14.79	*t* = 0.70	0.483
Median	120	120		
Range	100–150	100–150		
DBP (mmHg)				
Mean ± SD	75.33 ± 8.69	77.11 ± 8.43	*t* = 0.99	0.327
Median	70	80		
Rage	60–90	60–90		
Age of disease onset (years)				
Mean ± SD	30.96 ± 15.35			
Median	29			
Range	7–63			
Duration of disease (years)				
Mean ± SD	4.69 ± 4.89			
Median	4			
Range	1–30			
VASI score				
Mean ± SD	26.42 + 25.44			
Median	18			
Range	1–90			
Type	n (%)			
Acrofacial	7 (15.6)			
Focal	15 (33.3)			
Universalis	4 (8.9)			
Vulgaris	19 (42.2)			
VIDA score				
0	13 (28.9)			
1	4 (8.9)			
2	7 (15.6)			
3	10 (22.2)			
4	11 (24.4)			
VASI score				
Mean±SD	26.42 + 25.44			
Median	18			
Range	1–90			
Leucotricia				
Positive	3 (6.7)			
Negative	42 (93.3)			
Koebnerization				
Positive	6 (13.3)			
Negative	39 (86.7)			
Family history				
Positive	10 (22.2)			
Negative	35 (77.8)			

U, Mann-Whitney test; χ^2^, Chi-Square test; SD, Standard Deviation; SBP, Systolic Blood Pressure; DBP, Diastolic Blood Pressure; *t*: Student *t*-test; VIDA, Vitiligo Disease Activity; VASI, Vitiligo Area Severity Index.

a Significant.

The clinical data of vitiligo patients in this study was shown in Table 1.

### Lipid profile and blood sugar levels of the studied groups

There were significant high levels of cholesterol (207.16 ± 64.51 vs. 171.36 ± 38.05) and TG (143.84 ± 55.80 vs. 117.44 ± 27.44) as well as fasting (89.44 ± 15.93 vs. 80.11 ± 8.36) and 2 hours post prandial blood sugar (122.89 ± 27.10 vs. 106.00 ± 11.56) levels in vitiligo patients than controls (p < 0.001, p = 0.005, p = 0.001 and p < 0.001, respectively) (Table 2).

**Table 2:** Comparison between vitiligo patients and control group regarding lipid profile and blood glucose levels.

Variables	Patients (n = 45)	Controls (n = 45)	Test of significance	p-value
Cholesterol (mg/dL)				
Mean ± SD	207.16 ± 46.51	171.36 ± 38.05	*t* = 4.00	<0.001a
Median	208	165.7		
Range	121–300	114.0–270.9		
TG (mg/dL)				
Mean ± SD	143.84 ± 55.80	117.44 ± 27.44	*U* = 2.85^a^	0.005^a^
Median	140	117		
Range	35–268	70.9–111.0		
LDL (mg/dL)				
Mean ± SD	125.24 ± 49.40	110.18 ± 36.89	*U* = 1.64	0.105
Median	111	113		
Range	26–214	35.5–203.0		
HDL (mg/dL)				
Mean ± SD	42.27 ± 14.06	45.53 ± 4.90	*t* = 1.47	0.145
Median	40	45		
Range	13–105	32–57		
Fasting blood sugar (mg/dL)				
Mean ± SD	89.44 ± 15.93	80.11 ± 8.36	*t*=3.48	0.001^a^
Median	90	80		
Range	65–120	70–95		
2 h post prandial blood sugar (mg/dL)				
Mean ± SD	122.89 ± 27.10	106.00 ± 11.56	*t* = 3.85	<0.001^a^
Median	120	100		
Range	90–190	90–130		

SD, Standard Deviation; *t*, Student *t*-test; *U*, Man-Whiteny test; TG, Triglyceride; LDL, Low-Density Lipoprotein; HDL, High-Density Lipoprotein.

a Significant.

### MetS among vitiligo patients and the control group

MetS was significantly more prevalent among the studies vitiligo patients (13, 28.9%) than the control group (0%) (p < 0.001) (Table 3).

**Table 3:** Prevalence of MetS among vitiligo patients and the control group.

Variables	Patients (n = 45) n (%)	Controls (n = 4) n (%)	χ^2^	p-value
MetS				
Positive	13 (28.9)	0	15.20	<0.001^a^
Negative	32 (71.1)	45 (100.0)		

χ^2^, Chi-square test.

a Significant.

### FABP4 serum levels in vitiligo patients and the controls

**Figure 1:**
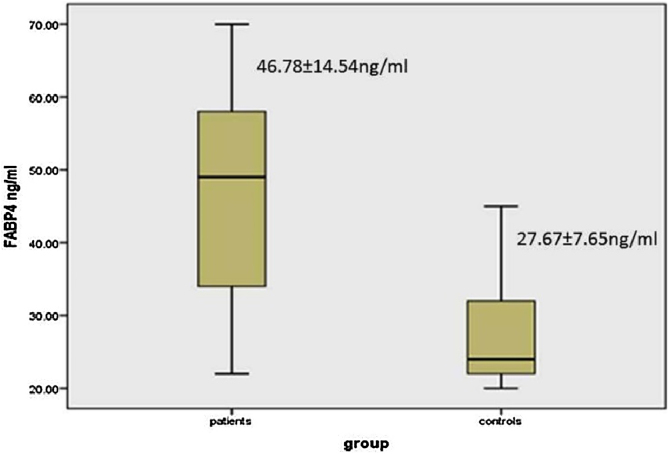
FABP4 levels in vitiligo patients and the control group.

There was a significant elevation of FABP4 serum levels in vitiligo patients (46.78 ± 14.54 ng/mL) than controls (27.67 ± 7.65 ng/mL) (p < 0.001) (Fig. 1).

### Role of FABP4 in early diagnosis of vitiligo

**Figure 2:**
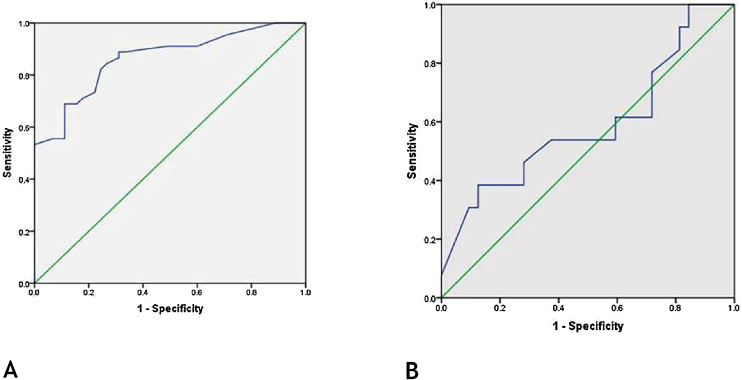
ROC of FABP4 levels for (A), early diagnosis of vitiligo [best cut off point 33.0 ng/mL, sensitivity of 82%, specificity of 76% and 0.863 area under the curve (p < 0.001)]. (B), detection of MetS in vitiligo patients [sensitivity of 77%, specificity of 28% and 0.590 area under the curve (p = 0.348)].

Receiver Operating Characteristic (ROC) curve showed that FABP4 level was a significant-good diagnostic test for early detection of vitiligo with best cut off point 33.0 ng/mL, the sensitivity of 82%, specificity of 76%, and 0.863 area under the curve (p < 0.001) (Fig. 2a).

### Role of FABP 4 level in the diagnosis of MetS in vitiligo patient

ROC curve showed that FABP4 was a poor diagnostic test to detect metabolic syndrome in vitiligo patients having 0.590 area under the curve (p = 0.34) (Fig. 2b).

### The relationship between FABP4 levels and studied parameters in vitiligo patients

In vitiligo patients, the high FABP4 serum levels were significantly associated with the presence of MetS (p = 0.037). However, FABP4 was not different according to the severity of vitiligo, as FABP4 serum level was not significantly correlated with VASI score (*r* = -0.21; p = 0.162) (Table 4).

**Table 4:** FABP4 levels in relation to VASI score and MetS in the studied vitiligo patients.

Variables	FABP4 (ng/mL) in patients (n = 45)	*t-*test	p-value
MetS	Mean ± SD		
Positive	55.85 ± 15.87		
Negative	45.53 ± 14.04	2.15	0.037^a^
VASI score	r		p-value
	−0.21		0.162

FABP4, Fatty Acid Binding Protein 4; SD, Standard Deviation; *t*, Student *t*-test; r, Spearman Correlation; VASI, Vitiligo Area Severity Index.

a Significant.

**Figure 3:**
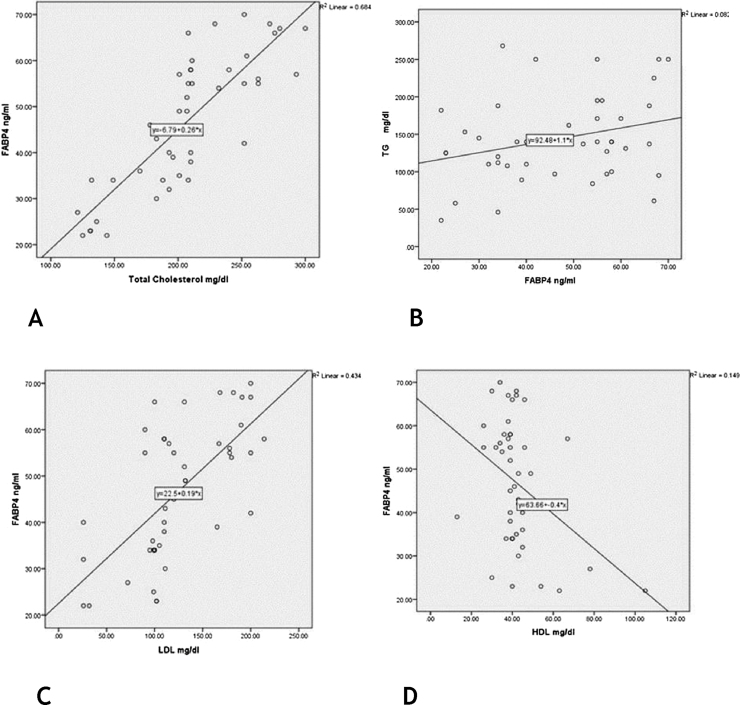
Correlations between FABP4 serum levels and lipid profile levels in vitiligo patients: (A), total cholesterol (*r* = 0.83; p = 0.001); (B), TG levels (*r* = 0.39; p = 0.047); (C), LDL (*r* = 0.66; p = 0.001); (D), HDL (*r* = -0.39; p = 0.009).

There were significant positive correlations of FABP4 serum levels with TG, cholesterol and LDL levels (*r* = 0.39; p = 0.047) (*r* = 0.83; p = 0.001) (*r* = 0.66; p = 0.001) respectively] and a significant negative correlation regarding HDL level (*r* = −0.39; p = 0.009) (Fig. 3).

## Discussion

In the present study, the authors investigated, for the first time, the possible role of FABP4 in vitiligo development through evaluation of its serum level in patients having NSV versus controls. The present study reported a significant increase in FABP4 circulating levels in vitiligo patients than matched peers, and these high concentrations were significantly associated with MetS in the studied cases, confirming that the pathogenesis of vitiligo has an immune-metabolic background.^18^

As the authors’ reported, Tanacan and Atkan^19^ revealed that the risk of developing MetS is increased in patients with NSV. The pathogenesis of vitiligo is not well known, but autoimmunity plus oxidative stress represents two important mechanisms responsible for vitiligo an etiopathogenesis.^20^ Oxidative stress is one of the main reasons for MetS development and can have a relation to the pathogenesis of some diseases like psoriasis and vitiligo.^11^ Additionally, an increased level of homocysteine, which is a tyrosinase inhibitor, may also be a contributing factor to the development of MetS in vitiligo patients.^21^

MetS increases the risk of developing diabetes mellitus type 2 and cardiovascular diseases by about 5 and 2 folds respectively.^22^ Therefore, the authors of the present study advocated that it is essential to prevent these associated serious complications of MetS by changing patients’ lifestyles. Additionally, optimal management of MetS may improve the clinical course of vitiligo.

FABP4 function has been linked to insulin sensitivity, lipid metabolism, and inflammation,^23^ as well as glucose production that contributed to the pathogenesis of immune-metabolic diseases.^24^ In agreement with these data, the authors reported a significant state of hyperglycemia and dyslipidemia in vitiligo cases, that was significantly associated with high FABP4 serum levels (significant positive correlations with TG, cholesterol, and LDL levels, and a significant negative correlation regarding HDL level).

Therefore, the present study suggested that FABP4 may have an active role during the disease process of vitiligo that could be mediated through associated dyslipidemia and hyperglycemia.

As vitiligo is considered one of the inflammatory diseases, FABP4 may act at the interface of inflammatory and metabolic pathways.^25^

FABP4 induces inflammatory responses through activation of the IκB Kinase-Nuclear Factor-kabba B (IKK-NF-κB) and jun N-terminal Kinase- Activator Protein-1 (JNK-AP-1) pathways,^26^ as well as for Tumor Necrosis Factor-alpha (TNF-α).^27^ The effect of TNF-α on cultured human skin melanocytes plays important role in vitiligo through NF-κB activation.^28^

The aberrant activation of innate immune cells in the skin of vitiligo patients includes inflammatory dendritic cells that migrate from the skin to draining lymph nodes presenting melanocyte antigens to T-cells and activate them. These cells also secrete cytokines which recruit and stimulate auto-reactive T-cells and then kill melanocytes directly.^29^ The FABP4 in dendritic cells has been shown to regulate T-cell priming.^30^

The auto-reactive tissue-resident memory T-cells inhibit melanin production and affect the regeneration of melanocytes by blocking regulatory T-cells locally.^31^ Recent outcomes confirm the presence of auto-reactive CD8^+^ cells with CD103^+^CD69^+^CD49a^+^ T_RM_ phenotype within the skin of vitiligo patients. The auto-reactive tissue-resident memory CD8+ demonstrates overexpression of FABP4 in vitro studies.^32^

Regarding the role of endothelial cells in vitiligo, human dermal microvascular endothelial cells secrete copious amounts of clusterin. This clusterin, through paracrine crosstalk between endothelial cells and melanocytes, can inhibit melanogenesis.^33^ The FABP4 has a potential role in endothelial cell growth by promoting cell proliferation, migration, survival, and morphogenesis.^34^

Based on the mentioned data, the authors postulated that the role of FABP4 in vitiligo pathogenesis could be mediated not only through its metabolic function (demonstrated hyperglycemia and dyslipidemia) but also via its immune-mediated mechanisms including up-regulated inflammatory cytokines,^27^ over-expressed auto-reactive tissue-resident memory T-cells^32^ and stimulated endothelial cells.^34^

FABP4 acts as an important mediator in the crosstalk between adipocytes and macrophages in adipose tissue. FABP4 knock-out mice were protected from the development of obesity, insulin resistance, and impaired glucose tolerance, and their adipocytes showed reduced lipolysis.^35^ In line with their findings, the authors observed a significant association between high FABP4 levels and the presence of MetS in present study's vitiligo patients. Also, Terra et al.^36^ found a relationship between circulating FABP4 levels and the presence of obesity and MetS.

In this study, although the present study demonstrated that FABP4 cannot predict MetS development in the studied vitiligo patients (that may be attributed to small sample size in this work), the authors revealed that FABP4 level was a significant good diagnostic test for early detection of vitiligo that could help in the diagnosis of vitiligo in confusing cases. Large-scale studies are needed to confirm this result.

## Conclusions

FABP4 may play an active role in vitiligo development and its targeting may have an appraising effect in clinical application in vitiligo management. The role of FABP4 in the disease process of vitiligo could be mediated through associated dyslipidemia and hyperglycemia. FABP4 may *be* a marker of vitiligo helping in its early diagnosis, but it does not appear to be useful for determining vitiligo severity, activity, or associated MetS.

## Study limitations

The small number of the studied subjects was the main limitation of the current study.

## Financial support

None declared.

## Authors’ contributions

Azza Gaber Antar Farag: Critical literature review; study conception and planning; approval of the final version of the manuscript.

Eman Abdelfatah Badr: Data collection, analysis and interpretation; approval of the final version of the manuscript.

Asmaa El-Shafey Soliman El-Shafey: Data collection; approval of the final version of the manuscript.

Mustafa Elsayed Elshaib: Statistical analysis; approval of the final version of the manuscript.

## Conflicts of interest

None declared.
